# Editorial: Advances in mitochondria-targeting in drug development for common disorders

**DOI:** 10.3389/fphar.2024.1520126

**Published:** 2025-01-03

**Authors:** Mundla Srilatha, Ganji Purnachandra Nagaraju

**Affiliations:** ^1^ Department of Biotechnology, Sri Venkateswara University, Tirupati, India; ^2^ School of Medicine, Division of Hematology and Oncology, University of Alabama at Birmingham, Birmingham, AL, United States

**Keywords:** mitochondria, melatonin, endoplasmic reticulum, pathological conditions, cystathionine γ-lyase, cardiovascular diseases

## Introduction

Mitochondria, commonly referred to as the cell’s power generators, play an essential role in regulating metabolism and apoptosis. Because of its fundamental importance in cellular health, maintaining mitochondrial integrity is necessary for the functioning of all organisms. In this Research Topic, we discuss all these links and current research reports on the interaction of mitochondrial membranes with the endoplasmic reticulum and the evidence that it represents a possible target in treating cardiovascular diseases.

Recent research has highlighted the potential effects of melatonin—a hormone known to regulate sleep—primarily on mitochondrial quality control mechanisms. This emerging area of research promises to open new therapeutic approaches for age-related diseases, neuropathology, and metabolism. Melatonin synthesized in the pineal gland has attracted attention for its antioxidant properties and role in reducing oxidative stress (Lei et al.). Oxidative stress significantly contributes to mitochondrial dysfunction, impairing energy production and increasing cellular damage. By scavenging free radicals, melatonin not only protects mitochondrial membranes but also increases the activity of the electron transport chain. This protective effect suggests that melatonin plays a vital role in promoting mitochondrial biogenesis and reducing apoptosis.

An interesting aspect of the effect of melatonin on mitochondrial quality control is its effect on mitochondrial fission and fusion efficiency. These mechanisms are important for maintaining mitochondrial shape and function (Lei et al.). Disruption of the balance between mitochondrial fission and fusion can lead to mitochondrial fragmentation, aberrant cellular division, and various ensuing diseases. Studies suggest melatonin may help restore this balance, improving mitochondrial function and cell turnover. Moreover, melatonin’s role in mitophagy—selective clearance of damaged mitochondria—offers an additional complement.

Ding et al. discuss all these links and current research reports on the interaction of mitochondrial membranes with the endoplasmic reticulum and the evidence that it represents a possible target in treating cardiovascular diseases. On the other hand, extensive research on patients with similarities in their effect of viral infection in most cases within and animal models has already shown that such a condition exists and how it manifests in the body. Mitochondria-associated endoplasmic reticulum membranes (MAM’s) atrophies – inducible by expression of specialized constructs – could not help the primary myocardium. Such alterations could be treated before becoming pathological conditions.

Disruption of these interactions has been linked to various cardiovascular conditions, making MAMs an intriguing focus for therapeutic intervention. The emerging connection between MAMs and cardiovascular pathology underscores the importance of mitochondrial quality control. In this circumstance, melatonin has been established for its determination along the mitochondrial run, notably its antioxidant properties and power to palliate aerobic focus.

Another promising avenue of research involves cystathionine γ-lyase (CSE), an enzyme known to release hydrogen sulfide (H_2_S) in response to cellular stress (Xiao et al.). New findings advise that the CSE inhibits mitochondrial aerobic focus in the Akt/Nrf2 sign tract (Xiao et al.). This connection highlights the protective role of H_2_S in mitochondrial health and positions CSE as a potential therapeutic target for combating oxidative damage in cardiovascular problems. Further expanding our understanding of the genetic underpinnings of cardiovascular diseases, recent studies have utilized mitochondria-associated genome-wide Mendelian randomization to identify causal genes in atrial fibrillation (Chen et al.). This modern access falls along the hereditary factors conducive to this current arrhythmia and highlights the implication of mitochondrial run in cardiovascular health.

The intersection of these Research Topic paints a compelling picture of how mitochondrial dynamics can aid in designing advanced therapeutic strategies for maintaining cardiovascular diseases. Targeting MAMs by their inhibitors enhances the use of protective influences in pathological conditions like cardiovascular disease. In summary, a deeper comprehension of the MAMs proposal is vital to propose the best strategies for blocking and healing cardiovascular diseases.

## Conclusion and future directions

In conclusion, the future of cardiovascular therapeutics may well hinge on our ability to harness the potential of mitochondrial biology. Melatonin, an antioxidant and alternator of mitochondrial function, seems to be an encouraging tackle for treating several mitochondrial quality control-associated diseases and injuries. CSE is an enzyme that yields H_2_S inside the mitochondria under stress conditions, partially progresses angiogenesis, and shows antioxidant effects. Therefore, the CSE^D187A^ mutant may be a possible target for therapy. MAMs mechanisms in cardiovascular diseases are vital for finding novel therapeutic targets for inhibiting or treating cardiovascular diseases. Interdisciplinary studies are essential to translate these understandings into clinical practice, ensuring we can potentially target the mitochondrial underpinnings of cardiovascular health.

